# A Review of Contemporary and Future Pharmacotherapy for Chronic Heart Failure in Children

**DOI:** 10.3390/children11070859

**Published:** 2024-07-16

**Authors:** Bibhuti B. Das

**Affiliations:** Department of Pediatrics, Heart Center, Mississippi Children’s Hospital, University of Mississippi Medical Center, 2500 North State Street, Jackson, MS 39216, USA; bdas@umc.edu; Tel.: +1-601-984-5250; Fax: +1-601-984-5283

**Keywords:** chronic heart failure, heart failure in children, HF with reduced ejection fraction (HFrEF), HF with preserved ejection fraction (HFpEF), pharmacotherapy for HFrEF and HFpEF in children

## Abstract

This review delves into the most recent therapeutic approaches for pediatric chronic heart failure (HF) as proposed by the International Society for Heart and Lung Transplantation (ISHLT), which are not yet publicly available. The guideline proposes an exhaustive overview of the evolving pharmacological strategies that are transforming the management of HF in the pediatric population. The ISHLT guidelines recognize the scarcity of randomized clinical trials in children, leading to a predominance of consensus-based recommendations, designated as Level C evidence. This review article aims to shed light on the significant paradigm shifts in the proposed 2024 ISHLT guidelines for pediatric HF and their clinical ramifications for pediatric cardiology practitioners. Noteworthy advancements in the updated proposed guidelines include the endorsement of angiotensin receptor-neprilysin inhibitors (ARNIs), sodium-glucose cotransporter 2 inhibitors (SGLT2is), and soluble guanylate cyclase (sGC) stimulators for treating chronic HF with reduced ejection fraction (HFrEF) in children. These cutting-edge treatments show potential for enhancing outcomes in pediatric HFrEF. Nonetheless, the challenge persists in validating the efficacy of therapies proven in adult HFrEF for the pediatric cohort. Furthermore, the proposed ISHLT guidelines address the pharmacological management of chronic HF with preserved ejection fraction (HFpEF) in children, marking a significant step forward in pediatric HF care. This review also discusses the future HF drugs in the pipeline, their mechanism of actions, potential uses, and side effects.

## 1. Introduction

In the United States, heart failure (HF) is estimated to impact between 12,000 and 35,000 children per year. Each year, HF-related hospitalizations account for about 11,000 to 14,000 admissions among children [1]. Although adult HF affects a larger number of adults, the impact of pediatric HF extends beyond mere statistics. Hospitalizations for pediatric HF are associated with longer stays, higher readmission rates, and increased hospital charges compared to adults. This is due to the frequent need for surgical or catheter-based interventions. Additionally, the demands of medical care can strain family structures and negatively impact parental economic productivity. When a child dies from HF, the economic consequences are magnified significantly due to the loss of potentially productive years. For these and other reasons, HF in children is a serious public health concern. This review paper explores the current landscape of pediatric HF management, highlighting unique considerations and emerging pharmacological innovations.

The latest ACC/AHA adult HF guidelines’ novel left ventricular ejection fraction (LVEF)-based classification system has influenced the proposed 2024 pediatric guidelines, albeit with modifications to suit the pediatric context where randomized controlled trial data are scarce [2]. The updated ISHLT guidelines propose detailed recommendations for managing chronic HF in children, focusing on HF with reduced ejection fraction (HFrEF) and HF with preserved ejection fraction (HFpEF), while omitting the HFimpEF category for its limited relevance in pediatrics. However, a classification scheme based solely on LV EF is limiting. A more innovative approach that incorporates new measures of ventricular performance, such as ventricular strain and accurate volumetric measurements, could better categorize HF patients. These parameters, however, have yet to be validated for use in children. The new classification based on LVEF is a step forward in defining pediatric HF for greater homogeneity and improved targets for clinical care and research.

Acute HF (within minutes to hours) [3] and chronic HF (progressive decline) [4] in children present distinct clinical challenges, with the former requiring immediate intervention and the latter necessitating long-term, quality-of-life-focused management. This review zeroes in on the pharmacological treatment of chronic HFrEF and HFpEF as per the latest proposal in the 2024 ISHLT guidelines, emphasizing the need for a holistic approach that considers the unique developmental and etiological aspects of HF in children. For further insights into the etiologies and diagnosis of pediatric HF, readers may refer to a related review article by the author [5].

## 2. Chronic HF with Reduced Ejection Fraction (HFrEF)

Heart failure (HF) is universally defined as a clinical syndrome characterized by symptoms and/or signs stemming from cardiac structural or functional abnormalities, confirmed by elevated natriuretic peptide levels or objective evidence of congestion [6]. In children, the ISHLT guidelines describe HF as a syndrome arising from ventricular dysfunction, volume, or pressure overload, leading to signs such as poor growth, feeding difficulties, respiratory distress, and fatigue, along with circulatory and neurohormonal changes [4].

Staging: The ACC/AHA staging system [2], integrated into pediatric HF guidelines since 2004, identifies patients at risk (Stage A) and those with early cardiac disease (Stage B), as well as those with symptomatic (Stage C) or advanced HF (Stage D). This system is crucial for a longitudinal perspective, differentiating between present and historical HF symptoms.

Classification: The ISHLT guidelines have been updated to encapsulate the essence of each classification system and scoring method, reflecting the latest research and its impact on pediatric HF care.

NYHA Classification: The NYHA classification, which ranges from Class I to IV, is a mainstay in adult HF assessment but is less applicable to pediatrics due to differing symptomatology in younger patients. The Ross Classification, designed for children, modifies the NYHA system to include symptoms prevalent in pediatric HF, providing a more pertinent evaluation tool [7].

Other Scoring Systems: The New York University Pediatric Heart Failure Index is another scoring system that accounts for clinical signs, symptoms, treatment, and ventricular anatomy [8]. Its wider adoption awaits further validation.

Global Rank End-point: The PANORAMA-HF trial [9] assessed ARNI versus enalapril in pediatric LV systolic dysfunction, introducing a global rank endpoint that combines clinical events, including a decrease in beta-natriurteic peptide (BNP) levels, improvement in NYHA/Ross classifications, and patient-reported symptoms.

## 3. Pathogenesis and Pathophysiology of Chronic HFrEF

**Figure 1:**
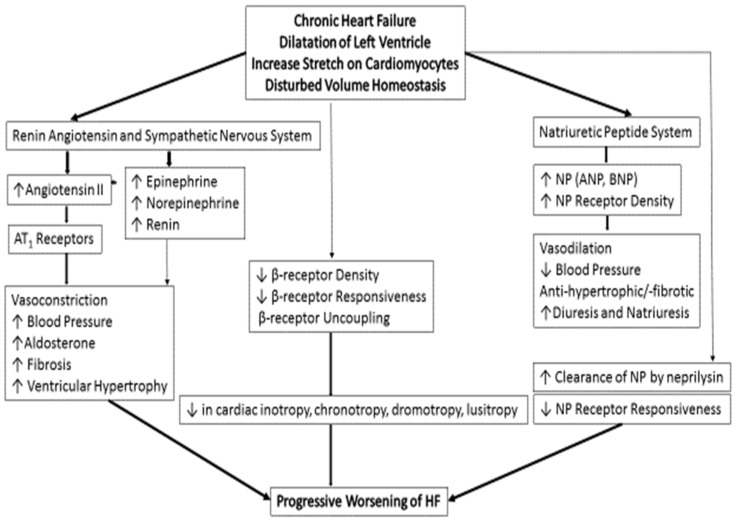
Pathophysiology of chronic HFrEF. ↓ Decrease, ↑ Increase. Reproduced from: Das, B.B. Children. 2018, 5(7), 88; [5] Creative Commons user license: https://creativecommons.org/licenses/by/4.0//.

Heart failure with reduced ejection fraction (HFrEF) often begins with an event that compromises cardiac output, triggering a series of adverse neurohumoral reactions (Figure 1). The resulting low cardiac output stimulates the sympathetic nervous system and the renin-angiotensin-aldosterone system (RAAS), causing increased peripheral resistance, heart rate, and myocardial contractility via β-adrenergic stimulation and cAMP elevation. The positive inotropic action is helpful to mitigate low cardiac output in the initial stage. However, persistent neurohumoral activation leads to pathological heart remodeling, such as promoting maladaptive cardiac hypertrophy and cell death. As this progression continues, heart efficiency declines, leading to the hallmark symptoms and complications of HF [10]. Treatment strategies for HFrEF focus on these underlying mechanisms, aiming to improve hemodynamics, inhibit detrimental neurohumoral responses, and prevent or reverse cardiac remodeling. It is important to note that the specific pathogenesis of HFrEF may vary according to individual patient factors and the root causes of the condition.

The clinical management of chronic HFrEF in children aims to reverse cardiac remodeling, ensure hemodynamic stability, and support growth and development. According to the proposed 2024 ISHLT guidelines, treatment may involve sequential or upfront combination therapy with the following drugs:

Diuretics: The intricate process of fluid retention and congestion in chronic HF is multifactorial, involving compensatory neurohormonal activation and fluid redistribution [11]. Diuretics are key to managing congestion symptoms in HF by inhibiting sodium reabsorption in the kidneys, thus promoting the excretion of salt and water. In adults, particularly the elderly, hospitalized for HF, loop diuretic therapy upon discharge has been associated with reduced 30-day mortality and HF readmission rates [12]. These findings support the class 1b recommendation for diuretic therapy in adults with fluid retention according to AHA/ACC adult HF guidelines [1]. For children, diuretic therapy is recommended for patients with fluid retention associated with ventricular dysfunction (HF stage C) to achieve an euvolemic state, although there is no direct evidence from trials indicating a survival benefit or reduced hospitalizations (Class I, Level of Evidence C).

Digoxin: Digoxin, a cardiac glycoside derived from the purple foxglove plant, was historically one of the initial treatments for HF and was widely used for symptomatic relief in these patients [13]. Recent insights suggest that while Digoxin is not commonly used as a first-line treatment for chronic HFrEF in children, it remains a part of the therapeutic arsenal, particularly in cases with fewer treatment options. Its role has been reaffirmed in certain pediatric conditions, such as improved interstage survival after the Norwood procedure for single ventricle anatomy [14]. However, the therapeutic window for Digoxin remains narrow, and the potential for arrhythmias because of altered serum potassium balance necessitates careful monitoring [15]. It is important to note that the landscape of pediatric HF treatment is evolving, with new drugs and therapies being explored to provide more tailored and effective care for young patients.

Renin-Angiotensin-Aldosterone System Inhibitors: For adults with HFrEF, core therapies include medications that inhibit the RAAS. This recommendation is backed by multiple randomized controlled trials (RCTs) showing significant improvements in morbidity and mortality with these medications [16]. In pediatric HF patients, there are no RCTs for angiotensin-converting enzyme (ACE) inhibitors, angiotensin receptor blockers (ARBs), or mineralocorticoid receptor antagonists (MRA). However, their use is recommended based on the positive outcomes observed in adult studies and the limited pediatric data available [17].

Angiotensin Receptor Neprilysin Inhibitors (ARNIs): ARNI is a combination of an ARB with a neprilysin inhibitor, which degrades natriuretic peptides [18]. Blocking neprilysin boosts the activity of various endogenous peptides, leading to vasodilation and anti-hypertrophic/anti-fibrotic effects, which counteract the harmful impacts of angiotensin, endothelin, and aldosterone. Additionally, inhibiting angiotensin synthesis is crucial because neprilysin inhibition alone can activate the RAAS, potentially due to angiotensin being a substrate for neprilysin. The PARADIGM-HF trial, which enrolled over 8000 adults with HFrEF and compared enalapril to ARNI, was halted after 27 months due to significant benefits in mortality and HF-related outcomes [19]. Consequently, ARNIs have received a Class 1 Level of Evidence A recommendation for adult HFrEF patients [1,20]. In pediatrics, the PANORAMA-HF trial did not meet its primary endpoint for improving a novel pediatric global HF rank endpoint [21]. However, an interim analysis showing improved natriuretic peptides at 12 weeks led to the Food and Drug Administration (FDA) and European Medicines Agency approving ARNI for children in 2019. According to proposed 2024 ISHLT guidelines, for children > 1 year with HFrEF, ARNI is a reasonable alternative to an ACE inhibitor/ARB (Class IIa, Level of Evidence B).

β-Adrenergic Receptor Blockers: The β-adrenergic receptor blockade aims to counteract the harmful effects of chronic sympathetic myocardial activation and reverse LV remodeling. Beta-blockers have significantly improved survival rates and reduced hospitalizations for HF in adults [22], earning them frequent use in managing pediatric HF (Class IIa, Level of Evidence C). However, a randomized controlled trial of Carvedilol in children with HFrEF did not demonstrate a difference in composite outcomes compared to the placebo [23]. Several factors may contribute to this discrepancy. The trial might have been underpowered and included a heterogeneous group of pediatric HF patients with varying systemic ventricle morphologies. Additionally, evidence now suggests age-related variations in beta-adrenergic receptor (AR) expression in the myocardium [24]. In children with idiopathic dilated cardiomyopathy (DCM), both beta-1 and beta-2 AR are downregulated, whereas in adults with DCM, only beta-1 AR shows downregulation [25]. A Cochrane review, including 7 studies with 420 children receiving β-blockers for HF, suggested a beneficial role of β-blockers in managing pediatric HF [26].

Ivabradine: Elevated heart rate is linked to worse outcomes in HFrEF [27]. Ivabradine targets channels in sinoatrial tissue, reducing the heart rate without affecting the sympathetic nervous system, and helped reduce HF hospitalization and death from HF in adults in the SHIFT and BEAUTIFUL trials [28]. A randomized trial showed Ivabradine’s benefits in reducing heart rate and improving LVEF in children with symptomatic chronic HF [29]. Ivabradine has been approved by the FDA for children over six months with symptomatic chronic HF who have persistent tachycardia despite an adequate dose of BB, with established dosing strategies across different age groups. Ivabradine is reasonable to use as additional therapy for children with stable HF where heart rate reduction is desirable (Class IIa, Level of Evidence B). Several commonly reported adverse effects include bradycardia, hypotension/hypertension, increased sensitivity to light, blurred vision, headaches, dizziness, and fatigue, among others. It is important to monitor these side effects and discuss them with parents and children.

Sodium-Glucose Cotransporter 2 Inhibitors (SGLT2i): The primary mechanisms underlying the clinical benefits of SGLT2i remain unclear. It is hypothesized to work by blocking the SGLT receptors in the proximal tubule of the nephron, which reduces the reabsorption of glucose and sodium into the bloodstream. This action leads to glycosuria and natriuresis, ultimately decreasing preload volume. These inhibitors improve ventricular loading conditions by reducing preload and afterload, which lowers blood pressure and enhances vascular function [30]. Additionally, SGLT2 inhibitors in HF regulate Na+/H+ exchange in cardiomyocytes, improve cardiac metabolism and bioenergetics, reduce necrosis and cardiac fibrosis (promoting reverse ventricular remodeling), and alter adipokine and cytokine production as well as epicardial adipose tissue mass [31]. In preclinical studies, SGLT2 inhibitors have demonstrated the ability to reduce the accumulation of uremic toxins, including p-cresol sulfate. This reduction decreases the need for renal detoxification. Additionally, SGLT2 inhibitors directly impact the kidneys by mitigating proximal tubule glucotoxicity and downregulating apical transporters (such as those for sodium, amino acids, and urate). These combined effects provide a metabolic basis for kidney and cardiovascular protection [32].

**Figure 2:**
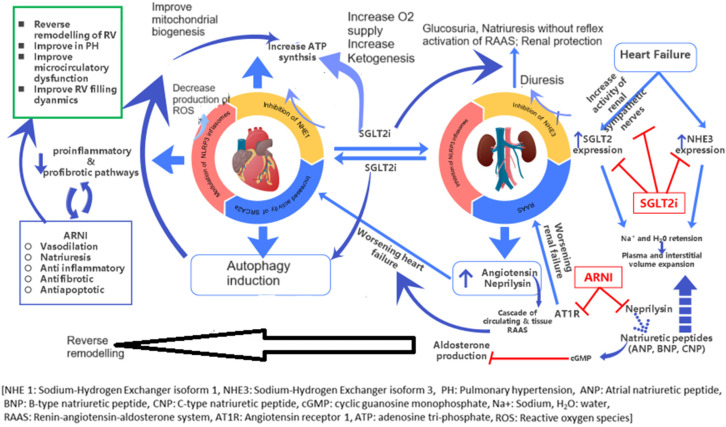
Mechanism of action of SGLT2i and ARNI to improve Chronic HFrEF.

Thus, SGLT2 inhibitors reduce preload and afterload and enhance metabolic pathways within cardiomyocytes, promoting efficient energy utilization by the heart (Figure 2). The clinical trial has been shown to improve mortality and HFrEF outcomes in adults with and without diabetes [33]. Furthermore, concomitant use of SGLT2i and ARNI has synergistic beneficial effect on chronic HFrEF [34]. While SGLT2is are classified as Class IIb with a level of evidence C for symptomatic HFrEF in children based on adult trials, their safety and tolerability in pediatric HF are supported by a limited case series [35]. When SGLT2 inhibitors are used in children with HF, it is important to monitor for adverse effects, including dehydration, urinary tract infections, genital infections, hypoglycemia, diabetic ketoacidosis, and hypotension, among others. SGLT2 inhibitors are generally contraindicated in children with Type 1 diabetes mellitus (T1DM) due to the increased risk of diabetic ketoacidosis.

**Figure 3:**
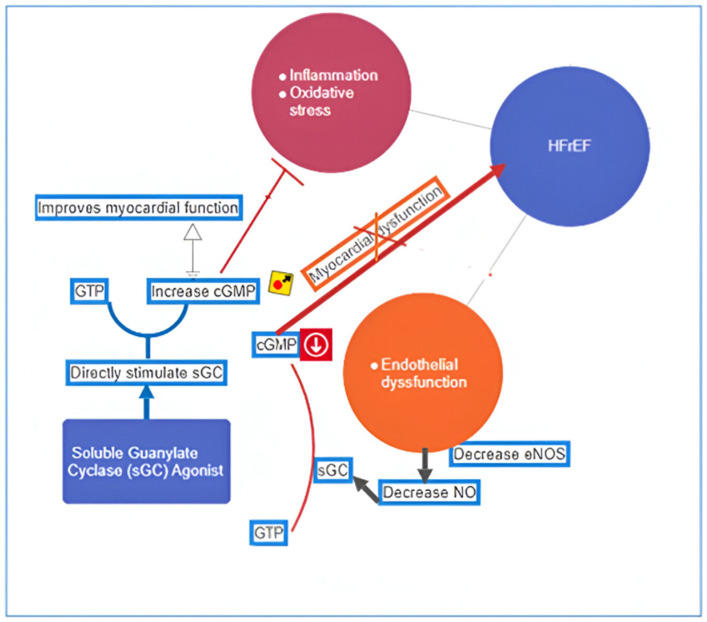
Mechanism of action of sGC stimulator (vericiguat) in HFrEF. [eNOS = endothelial nitric oxide synthetase, sGC = soluble guanylate cyclase].

Soluble Guanylate Stimulators (sGC): Vericiguat, a soluble guanylate cyclase (sGC) stimulator, has garnered attention for its potential in adult HF studies [36]. By modulating endothelial dysfunction, it impacts the nitric oxide (NO)-sGC-cyclic guanosine monophosphate (cGMP) pathway—a critical regulator of cardiovascular function (Figure 3). Endothelial cells produce NO, which diffuses to neighboring cells and binds to sGC. sGC then cleaves guanosine triphosphate (GTP) to form cGMP, acting as a secondary messenger. cGMP regulates vascular tone, cardiac remodeling, and myocardial contractility, influencing downstream targets like protein kinase G (PKG) and phosphodiesterases [37]. The VICTORIA study indicated a lower incidence of cardiovascular death or HF hospitalization with vericiguat [38]. Despite promising results in adults, vericiguat lacks published reports in pediatric HF. The ongoing Vericiguat in Pediatric Participants with Heart Failure Due to Systemic Left Ventricular Systolic Dysfunction (VALOR) trial investigates vericiguat’s efficacy, safety, and pharmacokinetics in stable systolic HF children [39]. Primary outcomes include NT-proBNP changes at week 16. The secondary outcomes include change from baseline to week 52 in log-formatted NT-proBNP, time to the first cardiovascular events such as cardiovascular death, hospitalization, or worsening of HF, adverse effects, and pharmacokinetic parameters for tablet and oral liquid formulations. Vericiguat represents a potential therapeutic option for children with HFrEF. As the VALOR study progresses, we await further evidence on its safety and efficacy. Vericiguat’s unique mechanism of action and promising results in adults make it an exciting candidate for pediatric HF management. Collaborative efforts between researchers, clinicians, and regulatory bodies are essential to advance vericiguat’s role in improving outcomes for children with HF. Despite challenges, the evolving landscape of pediatric HF holds promise. Novel therapies—such as ARNI, SGLT2 inhibitors, and sGC stimulators—offer hope for improved outcomes. It is important to monitor for side effects such as hypotension, anemia, dizziness, headache, nausea, and vomiting when using vericiguat for chronic HF.

The doses for commonly used HF drugs in children have been detailed in a previous review paper by the author, titled “Current State of Pediatric HF”, published in Children in 2018. In this review, the doses of newer HF drugs for children, where data are available, are summarized in Table 1.

**Figure 4:**
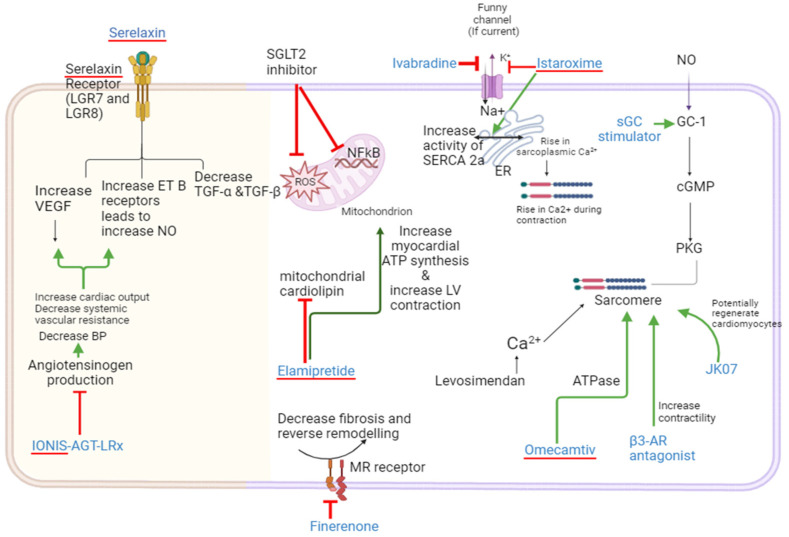
Mechanism of action of new investigational drugs for HF in the pipeline. sGC, soluble guanylate cyclase; cGMP, cyclic guanylate monophosphate; SGLT-2, sodium glucose co-transporter-2; ROS, reactive oxygen species; LGR, leucine-rich repeat-containing G-protein coupled receptor; NO, nitric oxide; NFκB, nuclear factor kappa B transcription factor; cGMP, cyclic guanylate monophosphate; SERCA, sarcoplasmic/endoplasmic reticulum Ca^2+^-ATPase; ATP, adenosine triphosphate; VEGF, vascular endothelial growth factor; ET, endothelin receptors; TGF, transforming growth factor; PKG, protein kinase G.

Future promising drugs for HFrEF in the pipeline: The landscape of HF treatment is witnessing rapid advancements, with ongoing research and clinical trials paving the way for innovative therapies. As we look to the future, several promising pharmacological agents are emerging for the treatment of HFrEF in adults. These experimental drugs represent the cutting edge of HF therapy, although their application in pediatric care remains unexplored due to the lack of data on children. Among the potential new treatments are Omecamtiv mecabril, Seralexine, Istaroxime, Ularitide, Elamipretide, Finerenone, APD418 (β3-Adrenergic Receptor Antagonist), IONIS-AGT-LRx, JK07, and others. As the field continues to evolve, these agents hold the promise of enhancing the management of HFrEF in adults. It is anticipated that with further research, including pediatric-specific studies, these therapies may one day be adapted for younger patients, offering hope for more effective treatment options. Figure 4 illustrates the mechanisms of several new HF drugs currently in the pipeline for future potential use.

Omecamtiv mecarbil, an innovative selective cardiac myosin activator (also referred to as a cardiac myotrope), is currently under development for potential use in treating HFrEF. It specifically binds to the cardiac myosin protein, facilitating the release of ADP-P from the myosin-actin-ATP complex. As a result, it increases the number of myosin heads that can attach to the actin filament, thereby enhancing cardiac sarcomere contractility. Importantly, systole duration is prolonged without elevating myocyte calcium levels or increasing myocardial oxygen consumption, unlike vasodilatory agents [40]. Notably, the GALACTIC-HF trial demonstrated a reduced incidence of composite HF events or cardiovascular-related deaths in adults with HFrEF who received Omecamtiv mecarbil compared to those who received a placebo [41]. Currently, there is no data on the efficacy of this drug in children, but it may be a promising alternative for improving outcomes in pediatric HFrEF. Commonly reported adverse effects may include dizziness, fatigue, exacerbation of HF, and myocardial infarction.

Serelaxin, a recombinant human relaxin-2, has vasodilatory properties and protects end-organ function. The RELAX-AHF trial showed it reduced worsening HF symptoms and possibly mortality at 180 days in hospitalized adults [42]. However, the subsequent RELAX-AHF-2 trial, with over 7000 patients, did not demonstrate benefits in mortality or rehospitalization rates [43]. A related pediatric phase II trial, RELAX-PEDS-PK, was halted following these results.

Istaroxime, a novel therapeutic agent under phase II trials, emerged in 2004 as a potential treatment for chronic HF. This synthetic compound, inspired by digitonin’s structure, exhibits dual action: it enhances cardiac contractility and relaxation. Its dual mechanism involves suppressing the Na^+^/K^+^ ATPase channel on the cell membrane [44] and promoting the activity of Sarcoplasmic/Endoplasmic Reticulum Ca^2+^-ATPase (SERCA) 2a in the sarcoplasmic reticulum [45]. Consequently, this leads to a rise in cytoplasmic calcium during heart contraction and a boost in calcium reabsorption during relaxation [46]. Compared to digoxin, Istaroxime shows a stronger contractile effect with a reduced risk of arrhythmias. Initially designed for intravenous use in acute heart failure, the HORIZON-HF study indicated that Istaroxime lowers pulmonary capillary wedge pressure and improves diastolic function after a 6-h infusion in patients [47]. As of now, its effects on the pediatric population remain unexplored. It is important to monitor side effects such as decreased heart rate, increased systolic blood pressure, nausea, and vomiting when Istaroxime becomes available for use in HF. Currently, no data are available for children.

Ularitide, a synthetic analog of the naturally occurring urodilatin peptide, plays a role in managing renal sodium absorption and maintaining water balance. Phase II trials in adults with acute decompensated heart failure (ADHF) have shown that Ularitide significantly lowers pulmonary capillary wedge pressure (PCWP) and alleviates symptoms of shortness of breath [48]. Further research involving 221 patients with acute HF revealed that Ularitide consistently decreased PCWP and increased stroke volume across various dosing regimens [49]. The TRUE-AHF trial, a phase III study, is examining its efficacy in a larger adult ADHF cohort [50]. Currently, there is no available data on the use of Ularitide in the pediatric population. Ularitide has several potential side effects, including hypotension, headaches, dizziness, nausea, and vomiting.

Elamipretide is a novel tetrapeptide that specifically targets mitochondrial cardiolipin [51]. By doing so, it aims to bolster mitochondrial function and, by extension, cardiac health. Pre-clinical studies have shown promising results, with Elamipretide significantly enhancing LV function, boosting myocardial ATP synthesis, and averting pathological LV remodeling in various animal models [52,53]. Despite these encouraging pre-clinical outcomes, clinical trials have not yet demonstrated a marked improvement in both HFrEF and HFpEF patients treated with Elamipretide [54]. This discrepancy may stem from several factors, including the relatively brief duration of Elamipretide therapy in human trials compared to the longer-term and higher-dose exposure in animal studies [55]. When Elamipretide is used for mitochondrial diseases, commonly reported side effects include headache, dizziness, abdominal pain, and flatulence, along with other unknown adverse events.

Finerenone is a novel, non-steroidal, selective MRA currently under investigation. It has demonstrated the ability to counteract many of the adverse effects associated with mineralocorticoid receptor overactivation [56]. Finerenone is in Phase III clinical trials for the treatment of HF. Following studies that highlighted its cardiovascular benefits for patients with chronic kidney disease (CKD) and type 2 diabetes mellitus (T2DM), Bayer is now exploring its potential to slow CKD progression in patients without T2DM. The FIND-CKD study, a multicenter, randomized, double-blind, placebo-controlled Phase III trial, is assessing the mean rate of change in the estimated glomerular filtration rate (eGFR) slope from baseline to 32 months [57]. Finerenone, when used for conditions related to T2DM, has several potential side effects. Commonly reported adverse effects include hyperkalemia, hyponatremia, hypotension, diarrhea, confusion, nausea, vomiting, irregular heartbeats, and numbness or tingling in the hands, feet, and lips.

IONIS-AGT-LRx is an investigational antisense medicine, conjugated with a ligand, designed to lower angiotensinogen production and thereby reduce blood pressure in patients with treatment-resistant hypertension. It is currently in Phase II clinical trials for the treatment of chronic HFrEF [57].

JK07 is a neuregulin-1 fusion antibody drug that has demonstrated regenerative potential in both large and small animal models of HF, including HFrEF and HFpEF. JK07 (SalubrisBio, Gaithersburg, MD, USA) is currently undergoing a Phase 1 trial (NCT04210375) to assess its safety, pharmacokinetics, and activity in patients with HFrEF [57].

Β3-adrenergic receptor (AR) antagonist (APD418) is a relatively new area of research in the context of HF. Unlike β1- and β2-ARs, which are the predominant adrenergic receptors in the heart, β3-ARs have unique characteristics. They lack the phosphorylation sites for G-protein-coupled receptor kinase (GPCRK) and do not have a protein kinase A (PKA) consensus sequence at the cytosolic C-terminal domain of the β3-AR. APD418 is a first-in-class investigational β3 AR antagonist that increases the contractility of human ventricular trabeculae from HFrEF donors [58]. The drug has been granted Fast Track designation by the FDA in 2020 and is currently in the Phase II stage of development.

## 4. Heart Failure with Preserved Ejection Fraction (HFpEF)

HFpEF is characterized by an LVEF of 50% or higher, accompanied by clinical indicators and symptoms of HF, as well as evidence of LV diastolic dysfunction. The American Society of Echocardiography (ASE) guidelines for evaluation of diastolic dysfunction in adults included four variables: (1) e′ velocity, (2) E/e′ ratio, (3) LA volume indexed to body surface area (LAVI), and (4) tricuspid regurgitation peak velocity (TRpV) [59]. The diagnostic criteria for HFpEF in adults may not directly apply to the pediatric or congenital heart disease (CHD) populations. In children, a comprehensive diagnosis of HFpEF should encompass a detailed medical history—highlighting symptoms such as orthopnea, exertional dyspnea, fatigue, and feeding difficulties—alongside a physical examination that looks for signs like hepatomegaly, jugular venous distension, peripheral edema, and pulmonary rales. Diagnostic tools such as echocardiograms are essential, and additional assessments like natriuretic peptide levels, exercise stress tests, cardiac MRI, and right heart catheterization can provide valuable supplementary information.

The causes of HFpEF in children are vast [60]. Addressing comorbid conditions such as hypertension, arrhythmias, and obesity is crucial in the management of HFpEF in children. Caloric restriction and exercise are under-recognized interventions for improving outcomes. Ensuring atrioventricular synchrony is vital for optimizing ventricular filling. The pharmacological approach to treating pediatric HFpEF is not well-established, with current practices largely based on expert opinion and the adaptation of adult clinical trial outcomes [61]. Consequently, the routine use of certain medications, including ARBs, MRAs, and ARNI, is not typically recommended for children with HFpEF. In older adults (over 60 years of age), β-blockers are associated with a lack of survival benefit and a higher risk of HF hospitalization in patients with HFpEF, particularly when LVEF was >60% [62,63,64]. These data suggest exercising caution when using β-blockers in pediatric patients with HFpEF due to the potentially higher risk of poor outcomes. Phosphodiesterase inhibitors (PDEi) do not improve LV function, pulmonary artery pressure, exercise capacity, or quality of life in patients with HFpEF in multiple studies in adults with HFpEF [65,66,67]. The current evidence for PDEi is limited and does not support their use in pediatric HFpEF. To date, no medication classes have been proposed in the revised ISHLT pediatric HF guidelines except diuretics with class I and level of evidence Class C. Diuretics help decrease fluid volume and mitigate symptoms. However, clinicians must carefully adjust dosages to avoid the risks associated with preload dependency and potential reductions in cardiac output due to relative volume depletion.

**Figure 5:**
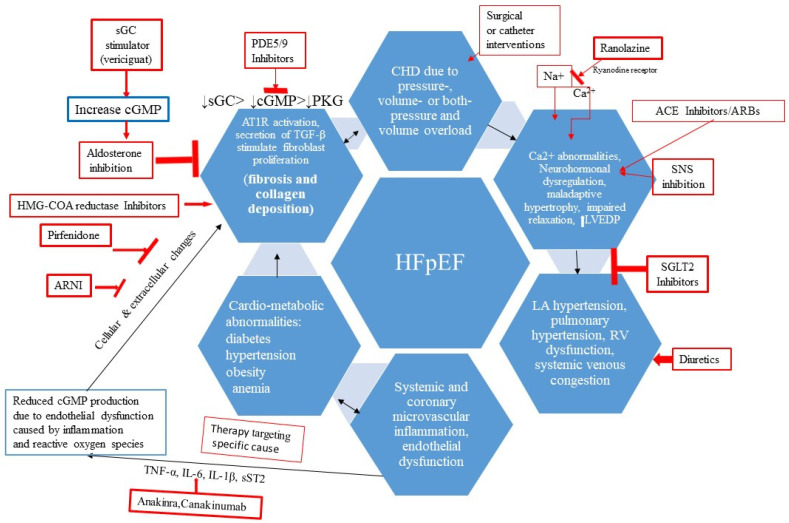
Newer drugs for HFpEF in the pipeline with their mechanism of action. TNF-α = tumor necrosis factor-α; IL-6 = interleukin-6; IL-1β = interleukin-1β; sST2 = soluble ST2 (receptor for IL-1 and 33); cGMP = cyclic guanosine monophosphate; PKG = protein kinase G; SNS = sympathetic nervous system.

Recently, SGLT2 inhibitors have emerged as promising drug therapies for reducing HF hospitalizations and deaths due to HF in HFpEF [68]. Recent updates to adult HF guidelines have introduced a Class I with Level of Evidence A recommendation for the use of SGLT2i in the treatment of HFpEF [69]. While there is a paucity of controlled studies on the use of SGLT2 inhibitors in the pediatric HF population, preliminary uncontrolled studies suggest potential benefits [35]. Given the high mortality associated with pediatric HFpEF, the consideration of SGLT2 inhibitors may be warranted as Class IIb with level of evidence C, though rigorous clinical trials are necessary to establish their safety and efficacy in children, especially those with CHD-related chronic HFpEF. There are many new drugs in the pipeline as potential therapeutic agents for HFpEF, as shown in Figure 5. The potential use of these advanced pharmacologic agents in pediatric HFpEF remains an area of exploration.

## 5. Conclusions

The management of chronic HF in children, including both HFrEF and HFpEF, often mirrors therapeutic strategies validated in adult populations. This parallel is drawn out of necessity due to the dearth of pediatric-centric clinical trials, leading to a reliance on consensus-based recommendations within ISHLT pediatric guidelines, which are categorized as Level C evidence. The inherent challenges in conducting pediatric HF trials—namely limited patient cohorts, diverse etiologies, and complex pathophysiology—suggest that these studies will likely continue to be underpowered. Consequently, it is anticipated that forthcoming pediatric HF guidelines will evolve primarily from safety and efficacy studies of treatments already established in adult HF research. However, the groundbreaking PANORAMA-HF trial [9] presents a potential shift in this paradigm, underscoring the imperative for future research to adopt cause-specific metrics, such as the measurement of NT- proBNP or BNP levels, paralleling the methodology of the seminal adult PARADIGM-HF trial [70]. Despite the instructive nature of adult HF trials in shaping pediatric drug research, direct application is impractical due to the fundamental disparities in disease processes and drug responses between children and adults. A sophisticated grasp of pediatric HF pathophysiology, combined with bespoke pharmacological strategies, is vital for the advancement of efficacious therapies. Incorporating pediatric-specific research findings into clinical protocols will significantly refine and amplify the impact of treatments for this susceptible demographic.

## Figures and Tables

**Table 1:** Dosages for newer and promising pediatric HF drugs.

Drug	Doses	FDA Approval
Ivabradine	0.02–0.05 mg/kg twice a day in <40 kg 25 mg twice a day in >40 kg	2019; off label use in children if tachycardia persists despite use of β-blockers
Sacubitril/Valsartan	Starting dose 1.6 mg/kg of the combined amount of both Valsartan and Sacubitril in <40 kg; increase every 2 weeks upward from 2.3 mg/kg up to a max dose of 3.1 mg/kg based on tolerance Valsartan 51 mg and Sacubitril 49 mg twice a day and titrate upward as tolerated in >40 kg	2019; in patients with symptomatic HF with LV dysfunction, over one-year-old
Dapagliflozin	0.1–0.2 mg/kg once daily, (Max 10 mg)	No approval: dose is determined empirically
Omecamtiv Mecarbil	No data	No data
Vericiguat	No data	No data

## Data Availability

Not applicable.
